# “Hell is other people”. Schizophrenia and urbanicity in the light of predictive coding theory

**DOI:** 10.3389/fpsyt.2025.1574944

**Published:** 2025-08-28

**Authors:** Alessio Mosca, Mauro Pettorruso, Giovanni Martinotti

**Affiliations:** Department of Neuroscience, Imaging and Clinical Sciences, “G. D’Annunzio” University, Chieti, Italy

**Keywords:** schizophrenia, self-disorders, predictive coding, urbanicity, psychosis

## 1 Introduction

The Predictive Coding theory (1) posits that the brain constantly generates predictions about the environment, allocating neural resources primarily to processing stimuli that violate these predictions (i.e., prediction errors), in order to update the brain’s predictive models of the environment. Sensory prediction is described as a hierarchical process, wherein neural activity encodes top-down expectations of sensory experiences, which are sent to lower-level brain regions. Any resulting discrepancies are then utilized to update internal models, facilitating the formation of new predictions (2, 3). This processing loop, known as the Predictive Coding framework, is based on the idea that the brain generates inferential models of the world to efficiently process the vast amount of incoming sensory information (2).

According to this model, impaired belief updating is thought to underlie the hallucinations and delusions characteristic of schizophrenia (4, 5). For example, an inappropriate overweighting of prior beliefs relative to incoming sensory information might lead to the perception of hearing intelligible words in response to ambiguous stimuli (6).

It is now well established that “urbanicity” -the impact of living in urban areas at a given time- constitutes a significant risk factor for the later development of psychosis and schizophrenia (7–9). A higher prevalence of schizophrenia in urban centers has been consistently confirmed (10, 11). Furthermore, the higher incidence of the disorder among migrant groups and ethnic minorities is one of the most consistent findings in schizophrenia epidemiology (12–14). Supporting this evidence, studies have observed a reduction in the risk of psychosis among children born in cities who later move to rural areas, as well as an increased risk among individuals raised in rural environments who move to cities during adolescence (15, 16).

With this article, we aim to propose an interpretation of the influence of urbanicity through the lens of the Predictive Coding model in the development of psychosis and schizophrenia. We suggest that the urban environment may shape predictive processes in the brain, potentially altering the balance between prior beliefs and sensory information. This perspective may offer new insights into the mechanisms underlying the increased risk of psychosis in urban settings and contribute to a more comprehensive understanding of schizophrenia.

## 2 Discussion

According to our hypothesis, urbanicity, a form of social stress (7), represents a form of overstimulation for individuals who are not accustomed to it. In such cases, internal predictive models may fail to generate coherent predictions of reality, potentially leading to the development of psychosis and schizophrenia.

Additionally, epidemiological studies have shown that environmental green spaces contribute to reducing the incidence of psychosis within the population (17–19).

From the perspective of the predictive coding model, it can be hypothesized that predicting reality is easier in a context with fewer stimuli, such as environments with ample green spaces, compared to densely populated, stimulus-rich urban areas. In other words, it appears to be easier to generate predictive models of reality in less reactive contexts. This may explain why green spaces act as a protective factor against the development of schizophrenia, whereas urbanicity is a significant risk factor, particularly for individuals who migrate from rural areas to high-density urban settings.

Converging experimental evidence indicates that exposure to natural environments modulates the neurobiological circuits governing the stress response, whereas urban living systematically imposes an “uncertainty load” on cognitive and biological systems. For instance, an fMRI study involving 63 healthy participants found that current urban residence was associated with heightened amygdala reactivity during a social−threat task; moreover, city dwellers showed stronger amygdala responses to unpredictable social evaluation, while an urban childhood engaged the perigenual anterior cingulate cortex—both regions pivotal for stress regulation (20). Complementing these findings, a randomized “one-hour walk” trial found that a single 60-min stroll in a forest, compared with a busy urban boulevard, significantly decreases amygdala activity, suggesting that even brief nature exposure can attenuate neural stress signatures (21). Epidemiologically, an analysis of 9,830 Chinese “new-generation migrants” revealed that migration from rural to urban areas is linked to higher levels of psychological distress (β = 0.305) and perceived stress (β = 0.328) than in peers who remained in rural settings, independent of socioeconomic covariates (22). Youth from disadvantaged urban neighborhoods display altered limbic reactivity when threat cues switch from predictable to unpredictable (23); irregular, impulsive traffic noise typical of metropolitan streets provokes stronger cortisol release and performance decrements than continuous noise (24); and life−history studies link the unsafe, chaotic conditions of low−SES urban settings with objectively measured environmental unpredictability and downstream stress−related psychopathology (25, 26). Taken together, these findings, demonstrating that moving from green rural settings to stimulus–rich urban environments increases stress vulnerability, also suggest, consistent with our hypothesis, that such a transition may elevate the unpredictability load on cerebral predictive mechanisms.

The increased likelihood of psychosis in migrants living in environments where they represent a minority also reflects the impact of perceived social stress (27), including social isolation and social defeat (13, 28).

Social capital is defined as “the set of resources, such as information, influence, and support, embedded in and accessible through social networks, sustained by trust and shared norms of reciprocity, that facilitate coordination and cooperation for mutual benefit” (29).

Multiple studies have demonstrated that social capital is markedly higher in rural than in urban settings, presumably because smaller, more homogeneous networks foster stronger, trust–based ties (30–32). Echoing this pattern, an inverse association between population density and social capital has been documented, indicating that less–dense, and therefore often smaller, communities tend to sustain higher levels of social capital (33). Elevated social capital has, in turn, been proposed to buffer the risk of psychosis, whereas social fragmentation and low social capital in urban neighborhoods may heighten that risk (11, 34).We hypothesize that smaller, traditional social structures may provide greater predictability and, consequently, lower stress levels compared to the urban social landscape, particularly for a migratory population accustomed to a more structured social life than the social disorganization found in urban neighborhoods.

Additionally, the sensory overload typical of metropolitan areas may compromise sensory gating, an automatic, pre−attentive inhibitory filter that dampens neural responses to repetitive or irrelevant input, (35, 36). Impaired gating exposes the brain to a flood of unfiltered information and can trigger aberrant salience, the inappropriate assignment of motivational significance to otherwise neutral stimuli (37). This process has been implicated in the emergence of delusional beliefs (38, 39). In line with our hypothesis, we propose that a state of “hypersalience” arises precisely when predictive systems fail to anticipate -or effectively model- the exceptionally high volume and unpredictability of urban sensory input, thereby overloading cortical prediction−error mechanisms. Within the predictive−coding framework, the sensory overload typical of urban settings weakens sensory gating (35), generating numerous, noisy prediction errors. To preserve perceptual coherence, the brain re−balances precision, down−weighting bottom−up evidence and conferring excessive confidence -hyper−precision- on higher−level predictions through dopaminergic modulation (4, 40). This imbalance drives aberrant salience, the motivational tagging of otherwise irrelevant stimuli (37), and stabilizes high−certainty hypotheses that withstand contradictory data, charting a computational path toward the formation of delusional beliefs (2, 39).

The phenomenological tradition has highlighted that self-awareness is inherently permeated by alterity (41), while empirical research has identified a basic pathology, described as a global alteration of subjectivity, known as self-disorders, which are central to schizophrenia (42, 43). Self-disorders involve disturbances in the dynamic structure of the first-person perspective, characterized by a fragile sense of self-presence and a tendency toward hyperreflexivity which implies active or passive modes of increased and intrusive self-awareness (44–46).

According to our hypothesis, the fragile sense of self-presence and the tendency toward hyperreflexivity, which disrupt the experience of alterity in schizophrenia, could be explained by a deficit in the ability to “predict” the behavior of others. In schizophrenia, this impaired ability to construct stable and coherent representations of social interactions leads to a sense of unpredictability and unfamiliarity in interpersonal contexts (47). Individuals with schizophrenia often struggle to anticipate the intentions, emotions, and actions of others, resulting in heightened anxiety and withdrawal from social environments (48). This impairment may manifest as difficulties in theory of mind, leading to misinterpretations of others’ behavior and contributing to delusional ideation (48, 49). Ebisch et al. (50) have already hypothesized that self-processing plays a role in mediating the beneficial effects of green spaces on psychosis. Moreover, we hypothesize that this failure in predictive social processing may contribute to the experience of ego-boundary disturbances, wherein individuals feel an abnormal permeability between the self and others (51). This can lead to phenomena such as thought insertion, passivity experiences, and delusions of influence, all of which reflect an altered sense of agency and a disrupted experience of self-other distinction (41, 48). Although intriguing, this proposal remains speculative and calls for rigorous empirical testing.

In this context, Sartre’s statement “Hell is other people” (52) gains new significance, emphasizing how encounters with others create anxiety in individuals with schizophrenia. The unpredictability of others -alterity- is known to be impaired in patients with schizophrenia (41).

Ultimately, from the perspective of predictive coding, a stressful factor can be understood as an event that is difficult to predict in relation to one’s internal predictive models of reality. When this “threshold of unpredictability” is exceeded, it can lead to psychopathological decompensation. In this sense, resilience may be defined as the ability to cope with events that are difficult to predict.

Ultimately, fear−related disorders such as PTSD are propelled mainly by hyper−reactive threat circuits and impaired fear−extinction mechanisms, systems in which prediction−error weighting is largely preserved. Schizophrenia, by contrast, hinges on a fundamental misallocation of precision between top−down predictions and incoming sensory evidence, a disturbance that clinically manifests as hallucinations and delusions. Predictive−coding failure thus stands as a cornerstone for understanding schizophrenia, even if further empirical work is required to refine and substantiate this framework.
